# Specifics of the Emotional Response of Patients Suffering From Major Depressive Disorder to Imagined Basic Tastes of Food

**DOI:** 10.3389/fpsyg.2022.820684

**Published:** 2022-02-07

**Authors:** Laura Jarutiene, Virginija Adomaitiene, Vesta Steibliene, Grazina Juodeikiene, Darius Cernauskas, Dovile Klupsaite, Vita Lele, Egle Milasauskiene, Elena Bartkiene

**Affiliations:** ^1^Psychiatry Clinic, Lithuanian University of Health Sciences, Kaunas, Lithuania; ^2^Department of Food Science and Technology, Kaunas University of Technology, Kaunas, Lithuania; ^3^Food Institute, Kaunas University of Technology, Kaunas, Lithuania; ^4^Institute of Animal Rearing Technologies, Faculty of Animal Sciences, Lithuanian University of Health Sciences, Kaunas, Lithuania; ^5^Department of Food Safety and Quality, Veterinary Academy, Lithuanian University of Health Sciences, Kaunas, Lithuania

**Keywords:** emotions, major depressive disorder, explicit methods, food-mood relation, imagined food tastes

## Abstract

Nowadays, the major depressive disorder (MDD) is a common disease that negatively affects the life quality of many people around the world. As MDD symptoms are closely related with the changes in food and eating, the relation between patients’ emotional responses and food tastes could be used as criteria for diagnostic. Until now, studies on the emotional response to different food tastes for patients affected by MDD have been poorly described in literature. Therefore, the aim of this study was to evaluate the emotional response of patients suffering from MDD to the imagined different food tastes and to compare the results with a control group. Emotional responses in tested participants were induced by using cards with words of basic food tastes such as “sweet,” “salty,” “bitter,” “sour,” and “neutral.” The assessment of emotional response was performed with FaceReader 6 software. The outcome of this study showed that participants with MDD expressed lower “happy” and “contempt” and higher “surprised” emotions, along with a higher negative valence mean, in comparison with controls for all tested basic tastes of food (*p* ≤ 0.05). When Likert scale was used, significant differences (*p* ≤ 0.001) in response were only found for “sour” and “salty” imaginary tastes between healthy group and patients with MDD. The findings of this study provide the additional data on food–associated emotion analysis of MDD patients and could be useful for the further development of the contactless method for early diagnosis of MDD.

## Introduction

Approximately 16% of the world’s population is affected by MDD (Liu et al., 2020). In many cases, the beginning of this disease remains unrecognized neither by doctors nor the patients. Therefore, the objective diagnosis of MDD in clinical settings requires the better understanding of the pathophysiology of MDD and creation of innovative testing methodologies.

Healthy diet and particular nutritional compounds might act as protective factors in the development, progression and treatment of depression (Gibson-Smith et al., 2020). It was reported that fish, vegetables, fruits, legumes, unprocessed meat, products with a high content of polyunsaturated fat and reduced content of fat or trans-fat were negatively related with depression, while sweetened drinks, candies and fast food were positively related with depressive symptoms (Lang et al., 2015; Gibson-Smith et al., 2020). Thus, the proper nutrition can maybe alleviate MDD in a sufferer. It was also found that diet and individual food products could have the effect on the occurrence, onset, severity, and duration of MDD (Roca et al., 2016). Other studies have shown that maintenance of a healthy diet reduces the severity of depressive symptoms (Rahe et al., 2014; Molendijk et al., 2018; Lassale et al., 2019).

Emotional reactions to food, reduced or increased appetite and loss of pleasure in eating are one of the factors predicting the development of MDD (Bartkiene et al., 2018). However, studies on the emotional responses of those affected by MDD to food taste are still very scarce. Emotions are psychological states brought on by neurophysiological changes, variously associated with thoughts, feelings, behavioral responses and a degree of pleasure or displeasure. Different emotions may increase or decrease food consumption quantities and meal frequency in the same person (Prefit and Szentagotai-Tãtar, 2018). There is information in the scientific literature that imagination and real tasting of food evoke similar emotions. Even seeing, smelling or thinking about food products elicit subjective feelings of craving as well as physiological responses (Haasova et al., 2016). Moreover, imagined odors could affect the taste perception in the same way as do perceived odors (Djordjevic et al., 2004).

Our previous study showed that the emotional response induced by different food tastes can be related to MDD and could be used as a diagnostic indicator of early stage MDD (Bartkiene et al., 2018). We found that the Noldus FaceReader technique is very promising and sufficiently accurate to detect differences in facial expressions induced by different food tastes for different mood groups; furthermore, this technique is more sensitive than the standard method of using a hedonic scale (Bartkiene et al., 2019). Moreover, this method is a contactless and therefore facilitates the testing procedure. In general, there are several ways to measure emotions elicited by food but explicit methods are the most prominent. Explicit methods thus remain a popular approach among practitioners in consumer and sensory research because they are quick to use, the data are easy to process and they are also user friendly (requiring little involvement by the participant) (Lagast et al., 2017). Nevertheless, studies mention major limitations and problems with explicit methods: they run the risk of being cognitively biased, which may affect the validity of emotion assessment; social desirability and self-representation biases can influence the explicit self-reported measures of emotion; they are retrospective because emotions are elicited after the experience; and some participants could lack the introspective capacity to correctly identify, recognize and verbalize the perceived emotion (Lagast et al., 2017). Implicit methods of measuring emotion avoid the limitations of explicit methods because are indirect, not self-reported and not under the conscious control of the consumer. Nowadays, these methods are only scantly explored in consumer and sensory research. Expressive measures, particularly the registration of facial expressions, are the most popular of the implicit methods. Explicit measures cover a large number of emotions whereas implicit measures generally cover a small number. Facial expression measurement is limited to the six basic emotions (Ekman, 1993) because it has proved to be unable to distinguish among a large number of different, especially positive, emotions. Some suggest a dimensional approach, such as valence and arousal dimensions, whereas others propose discrete emotions. Our studies examine (both explicitly and implicitly) the methodologies, measures and instruments used in consumer and sensory research to assess emotions induced by food in the context of food behavior.

It is hypothesized that, there will be differences in emotional response, measured with FaceReader, of patients with MDD and healthy people to imaginary basic tastes of food. Therefore, the aim of this study was to evaluate the emotional response of patients suffering from MDD to the imagined different food tastes and to compare the results with a control group (subjects without MDD for at least 1 year).

## Materials and Methods

### Assessment of Participants’ Emotions Elicited by Imagined Basic Tastes

**FIGURE 1:**
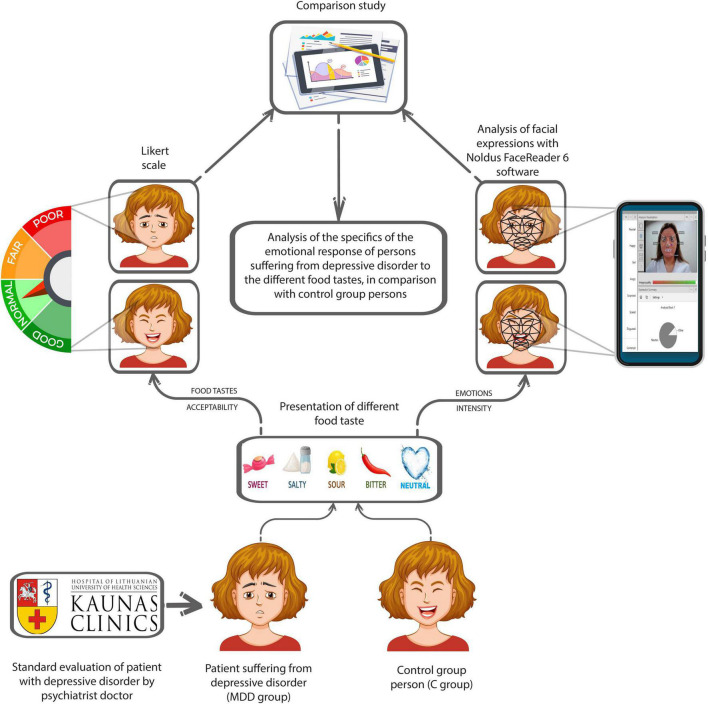
Principal scheme of the experiment (MDD – major depressive disorder).

The assessment of emotions was done with FaceReader 6 software (Noldus Information Technology, Wageningen, Netherlands). This software recognizes six universal expressions: “Happy,” “Sad,” “Angry,” “Surprised,” “Scared,” and “Disgusted.” Moreover, it identifies “Neutral” and “Contempt” states and calculates “Valence,” which describes person’s emotional state (positive/negative). The intensity of emotion is expressed as a value between 0 and 1 (−1 and 1 for “Valence”). The experimental scheme is given in Figure 1.

The study was performed at the Psychiatry Clinic of the Lithuanian University of Health Sciences (Kaunas, Lithuania), evaluating two groups of study participants: patients with diagnosis of MDD (DeDi group) and control group subjects without diagnosis of MDD for 1-year period (C group). Diagnosis of MDD was done by psychiatrist using diagnostic criteria of Diagnostic and Statistical Manual of Mental Disorders (DSM–5). The standard Montgomery and Asberg Depression Rating Scale (MADRS; Williams and Kobak, 2008) was used to assess the severity of MDD symptoms in patients and in controls. The overall acceptability of the imaginary basic tastes of food was assessed using a 10-point Likert scale from 0 (dislike extremely) to 10 (like extremely). The study procedures were performed in the morning after 8 h of fasting (water was allowed). Cards with various food tastes (“sweet,” “salty,” “bitter,” “sour,” and “neutral”) were presented to the tested person one by one. After each card, the person was asked to name a food product associated with the word on the card and to give a score on the line scale from 0 (dislike extremely) to 10 (like extremely) for how he/she likes the product and/or taste. Then, the person was asked to imagine how he/she tastes this food product. No timer was used for this. The procedure was recorded with a Microsoft LifeCam Studio webcam. The recordings were analyzed with FaceReader 6 software.

### Statistical Analysis

The data were analyzed using SPSS Version 24 software (SPSS Inc., Chicago, IL, United States). To study the different food-induced emotions of patients with and without MDD, 87 patients suffering from MDD and 87 control subjects were tested. Statistical differences with *p* values of ≤0.05 and ≤0.01 were considered to be significant and means were compared by Fisher’s least significant difference (LSD) *post hoc* test.

### Ethical Approval

The protocol of this study was approved by the Regional Bioethics Committee (No. 04/2017). All subjects were informed about the study using a “Personal Information Form.” Subjects were included in the study if they agreed to participate and signed the “Informed Consent Form.” The study was conducted in accordance with the guidelines of Good Clinical Practice and the principles of the Declaration of Helsinki.

## Results

### Emotional Response of People With and Without Major Depressive Disorder to Imagined “Sweet” Tasting Food

**FIGURE 2:**
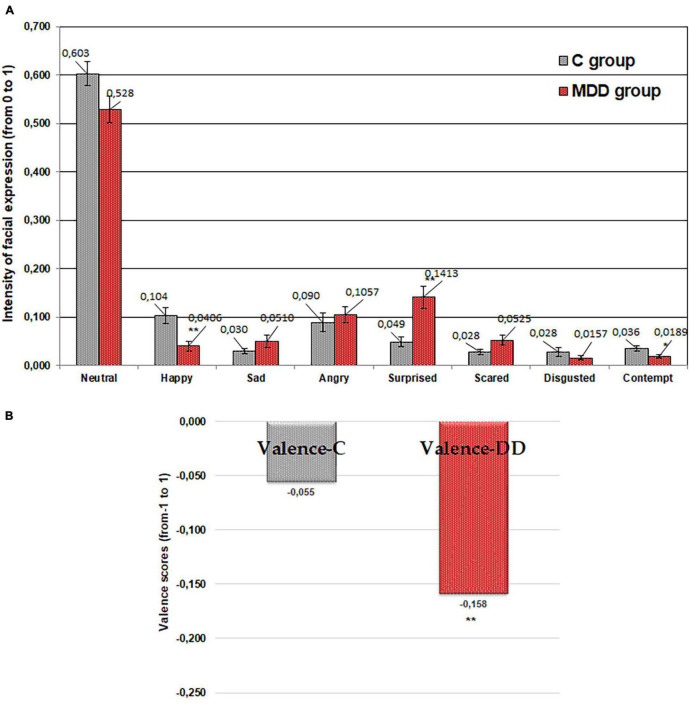
Emotions (“neutral,” “happy,” “sad,” “angry,” “surprised,” “scared,” “disgusted,” and “contempt”) induced by imagined “sweet” tasting food **(A)** and valence **(B)** for patients with major depressive disorder (MDD group) and without MDD (C group). Significant difference between groups: *p ≤ 0.05; **p ≤ 0.01.

A lower “happy” emotion was expressed by patients with MDD in response to “sweet” taste (average 60.9% lower; *p* ≤ 0.01) in comparison with the control group (Figure 2A). Furthermore, significantly lower (average 47.1% lower; *p* ≤ 0.05) “contempt” in patients with MDD was established in comparison with the control group. However, in contrast, a higher “surprised” emotion was expressed by patients with MDD (average 186.6% higher; *p* ≤ 0.01) in comparison with controls. Furthermore, the valence results showed that the valence mean correlates significantly with the emotional state of patients suffering from MDD (average 187.2%; *p* ≤ 0.05): a higher negative valence mean was established in those with MDD in comparison with controls (Figure 2B).

### Emotional Response of Patients With and Without Major Depressive Disorder to Imagined “Salty” Tasting Food

**FIGURE 3:**
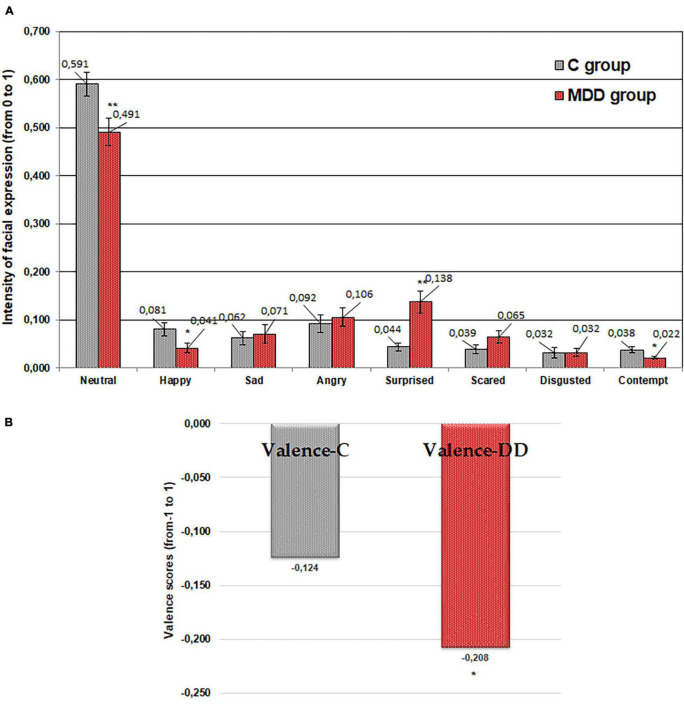
Emotions (“neutral,” “happy,” “sad,” “angry,” “surprised,” “scared,” “disgusted,” and “contempt”) induced by imagined “salty” tasting food **(A)** and valence **(B)** for patients with major depressive disorder (MDD group) and without MDD (C group). Significant difference between groups: *p ≤ 0.05; **p ≤ 0.01.

As for “sweet” food taste, similar tendencies were established for “salty” food taste (Figure 3). It was found that patients suffering from MDD expressed, on average, 16.9 and 48.6% lower (*p* ≤ 0.01 and *p* ≤ 0.05, respectively) “neutral” and “happy” emotions in comparison with the control group. Furthermore, statistically significant differences between patients with MDD and the control group for expression of “contempt” (43.2% lower, *p* ≤ 0.05) were found. In contrast, expression of “surprised” was higher in patients with MDD (212.8% higher; *p* ≤ 0.01) compared with the control group. Furthermore, patients with MDD showed, on average, a 67.7% (*p* ≤ 0.05) higher negative valence mean in comparison with the control group.

### Emotional Response of Patients With and Without Major Depressive Disorder to Imagined “Bitter” Tasting Food

**FIGURE 4:**
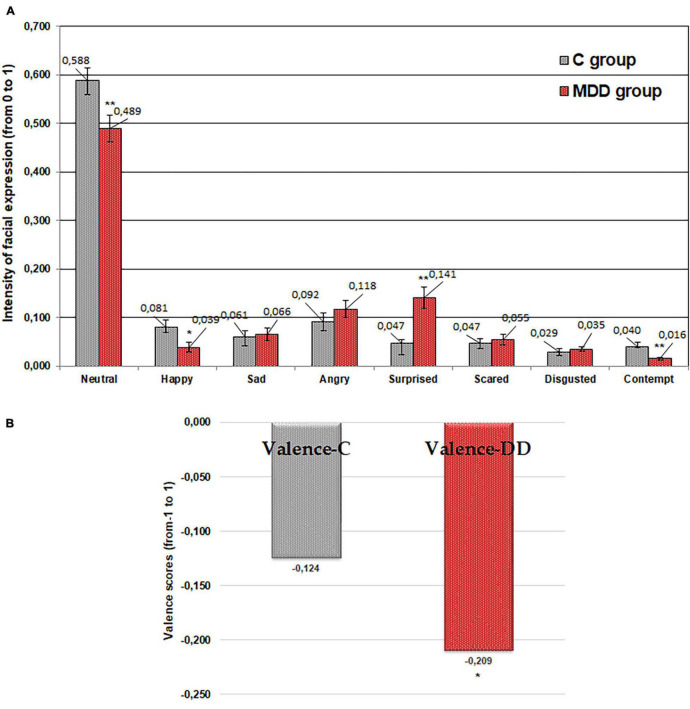
Emotions (“neutral,” “happy,” “sad,” “angry,” “surprised,” “scared,” “disgusted,” and “contempt”) induced by imagined “bitter” tasting food **(A)** and valence **(B)** for patients with major depressive disorder (MDD group) and without MDD (C group). Significant difference between groups: *p ≤ 0.05; **p ≤ 0.01.

Lower expression of “neutral,” “contempt,” and “happy” emotions by patients suffering from MDD was established for “bitter” taste [average 16.8% (*p* ≤ 0.01), 61.4% (*p* ≤ 0.01), and 51.6% (*p* ≤ 0.05) lower, respectively] in comparison with controls (Figure 4). In contrast, expression of “surprised” was higher in patients suffering from MDD (average 199.2% higher, *p* ≤ 0.01) compared with the control group. Furthermore, patients with MDD showed, on average, a 68.8% (*p* ≤ 0.05) higher negative valence mean in comparison with the control group.

### Emotional Response of Patients With and Without Major Depressive Disorder to Imagined “Sour” Tasting Food

**FIGURE 5:**
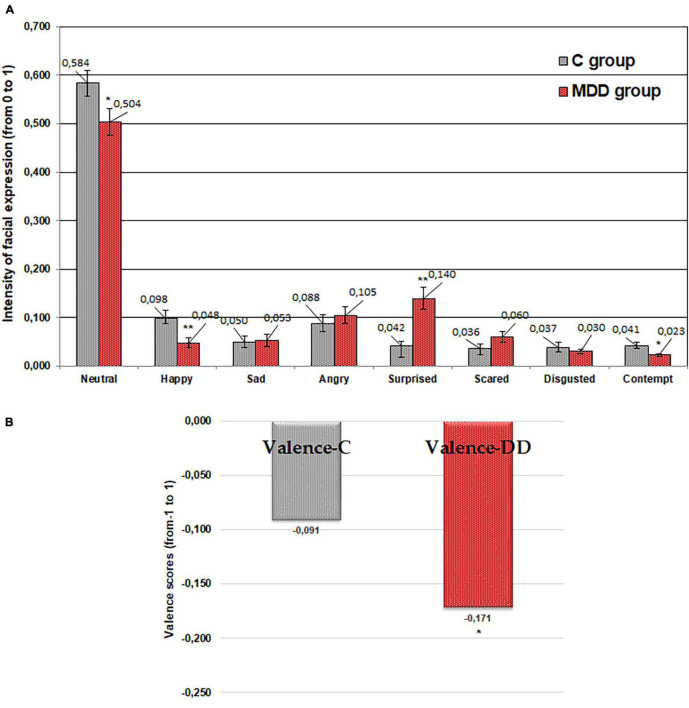
Emotions (“neutral,” “happy,” “sad,” “angry,” “surprised,” “scared,” “disgusted,” and “contempt”) induced by imagined “sour” tasting food **(A)** and valence **(B)** for patients with major depressive disorder (MDD group) and without MDD (C group). Significant difference between groups: *p ≤ 0.05; **p ≤ 0.01.

With regard to “sour” food taste, patients suffering from MDD expressed, on average, 13.6, 44.1, and 51.4% lower (*p* ≤ 0.05 and *p* ≤ 0.01) “neutral,” “contempt,” and “happy” emotions, respectively, in comparison with the control group (Figure 5). In contrast, expression of “surprised” was higher in patients with MDD (average 236.0% higher; *p* ≤ 0.01) compared with the control group. Furthermore, patients with MDD showed, on average, an 89.0% (*p* ≤ 0.05) higher negative valence mean in comparison with the control group.

**FIGURE 6:**
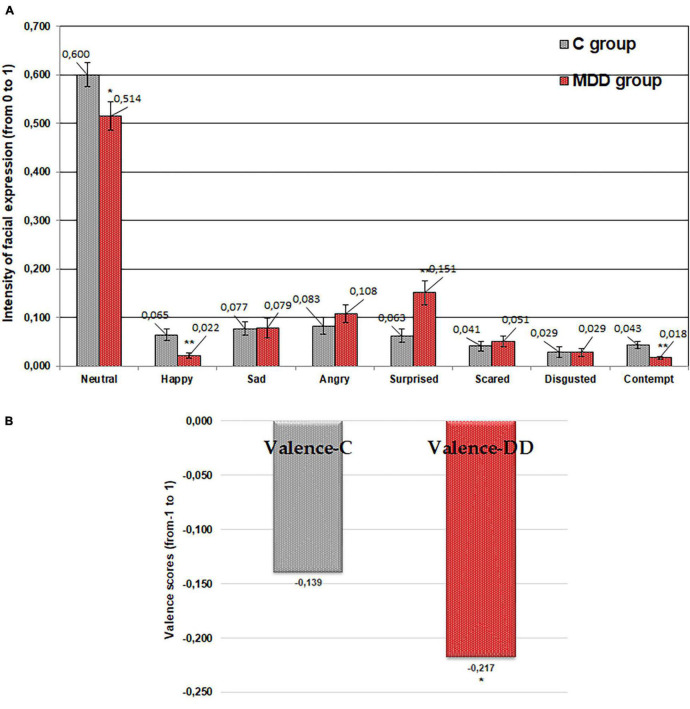
Emotions (“neutral,” “happy,” “sad,” “angry,” “surprised,” “scared,” “disgusted,” and “contempt”) induced by imagined “neutral” tasting food **(A)** and valence **(B)** for patients with major depressive disorder (MDD group) and without MDD (C group). Significant difference between groups: *p ≤ 0.05; **p ≤ 0.01.

It was found that patients suffering from MDD expressed, on average, 14.3, 59.1, and 65.8% lower (*p* ≤ 0.05 and *p* ≤ 0.01) “neutral,” “contempt,” and “happy” emotions, respectively, in comparison with the control group (Figure 6).

In contrast, expression of “surprised” was higher in patients with MDD (average 139.6% higher, *p* ≤ 0.01), compared with the control group. Furthermore, patients with MDD showed, on average, a 56.2% (*p* ≤ 0.05) higher negative valence mean in comparison with the control group.

### Acceptability of Imagined “Sweet,” “Salty,” “Bitter,” “Sour,” and “Neutral” Tasting Food Obtained Using a 10-Point Likert Scale

**FIGURE 7:**
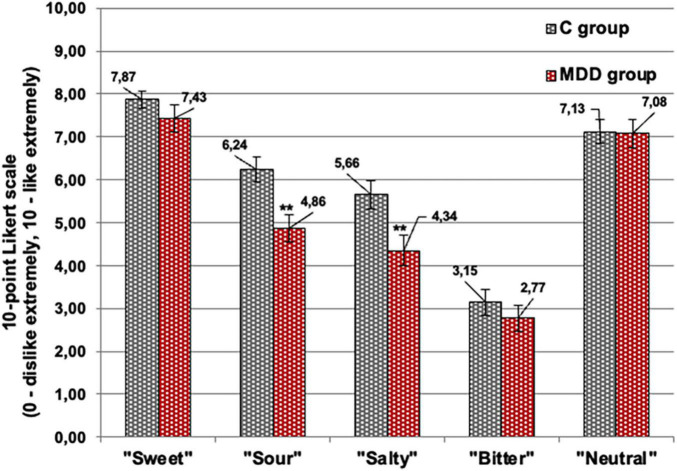
Acceptability of the imagined different food tastes (sweet, salty, bitter, sour, and neutral) evaluated using a 10-point Likert scale (0, dislike extremely; 10, like extremely). Significant difference between groups: **p ≤ 0.01 [patients with major depressive disorder (MDD group) and without MDD (C group)].

Evaluation of the emotional response to “sour” and “salty” tastes using a Likert scale showed, on average, 22.1 and 23.2% lower acceptability (*p* ≤ 0.001), respectively, for patients with MDD in comparison with the control group (Figure 7). However, other relations between the imagined food tastes and emotions induced for patients with MDD and the controls were not established.

## Discussion

According to a World Health Organization [WHO] (2017) report, MDD affects more than 300 million people around the world. MDD leads to neurotransmitter imbalance in the brain, pituitary–hypothalamic–adrenal axis dysfunction and changes of neurotrophic factor, all of which can lead to neurodegenerative/dementia disorders (Lebedeva et al., 2017). Nowadays, there is growing public interest in healthy nutrition and research has started to dig deeper into the influence of nutrition on mental health (Zepf et al., 2015); the influence of diet, as well as separate food products, on the course of MDD has been published (Roca et al., 2016). According to O’Neil et al. (2013) and Roca et al. (2016), newly developed effective public health nutrition strategies can work as preventive factors for MDD. However, as our previous study showed, many factors have the influence on food choice, including individual factors (gender, age, education, psychological characteristics, food eating habits, and food characteristics) (Guiné et al., 2020).

Usually, patients with MDD are characterized as having a lower appetite, change of food taste feeling and loss of enjoyment during food consumption, which are important diagnostic criteria for MDD. However, it should be pointed out that atypical MDD leads to a strong preference for high-energy foods, an uncontrolled appetite and eating satiation (Singh, 2014). It is commonly accepted that the emotional state of a person influences food choice and intake (Robbins and Fray, 1980; Ganley, 1989; Macht, 1999, 2008; Canetti et al., 2002). Therefore, persons suffering from MDD could show individual food choice but some of these characteristics could be related and specific exclusively to this group with this particular diagnosis.

The current study analyses a clinical population and in order to avoid any interventions, that could disturb patients’ health status and to simplify the whole testing procedure, the participants were asked to imagine the basic tastes of food instead of tasting actual ones. Furthermore, it would be significantly more difficult to gather a representative group of patients affected by MDD who would agree to taste different foods. Therefore, allergic reactions of patient to food are still possible, as well as unwanted change of the diets of the hospital created for them, and the resulting responsibilities did not allow us to choose a real food assessment and we decided to choose the senses of imaginative taste. According to literature, emotions elicited by imagination and real tasting could be similar. Imagined smell could influence the taste perception as the actual smelling (Djordjevic et al., 2004), while visualization of food products could evoke subjective feelings and physiological responses (Haasova et al., 2016).

The measurement of the emotions is a relevant issue for several decades. Twenty anatomically independent muscles in the human face are involved in the formation of facial expressions, which are highly related to emotions (Danner and Duerrschmid, 2018). There is a wide range of automatic facial expressions analysis (AFEA) systems available and most of them use similar steps of operating principle such as face acquisition, facial feature extraction and facial expression classification (Danner and Duerrschmid, 2018). AFEA is a very fast real-time analysis of facial expressions and results in high ecological validity. Moreover, the high accuracy in the classification of emotion and activation of specific action units can be reached by AFEA. In the study of Lewinski et al. (2014), FaceReader reached the facial action coding system (FACS) index of agreement of 0.67 and accuracy of 88% on average when classifying six basic emotions in two emotion data sets. The effectiveness of AFEA systems, including FaceReader, was already demonstrated in numerous studies related with food consumption and advertising research (Danner and Duerrschmid, 2018).

The outcome of performed study showed that the emotional responses to the imagined different food tastes in people suffering from MDD were significantly different from those in the control group. The significantly lower expression of “happy” and “contempt” emotions in patients with MDD for all food tastes was found. “Happy” is the only positive emotion and “contempt” is a standalone emotion that is often accompanied by anger, usually in mild form, such as annoyance. The decrease in these emotions could be explained by the mood changes in patients suffering from MDD, related to their apathetic state and inability to express not only positive emotion as “happy” but also negative emotion as “contempt.” The study of Yoon et al. (2016) showed that people with MDD and anxiety disorders have a distinctive difficulty with the recognition of facial expressions. Other study showed that moods increase the likelihood and intensity of “matching” emotions and decrease the likelihood and intensity of “opposing” emotions (Watson et al., 1988). Because of the dominative negative mood in depressed state, facial expressions related with negative valence emotions will intensify, while those related with positive valence emotions will weaken.

It is worth noting that the “surprised” emotion was statistically higher in patients with MDD compared with the control group for all the tested imagined food tastes. This can be explained by the fact that patients suffering from MDD have a variety of anxiety symptoms and any communication causes them higher anxiety and “surprised” emotion. In addition, this study showed that the valence mean correlates significantly with the emotional status of patients with MDD. The patients with MDD showed, on average, 187.2, 67.7, 68.8, 89.0, and 56.2% (sweet, salty, bitter, sour, and neutral, respectively; *p* ≤ 0.05) higher negative valence means in comparison with the control group. These results indicate that the emotional status of patients with MDD was more negative than in those without MDD.

Our previous study showed that evaluation of facial expressions using the FaceReader-4 technique could correlate with self-reported hedonic liking in persons not suffering from MDD (Juodeikiene et al., 2014). However, patients with MDD often find it difficult to assess their feelings by using a Likert scale. Alexithymia and impulsivity could be the elements that create the possible indirect pathways in the relation between depression, emotional, and external eating. Depression and alexithymia, in particular the alexithymic construct “difficulty identifying feelings,” are highly related (Parker et al., 1991; Ouwens et al., 2009). Therefore, we believe that the inability of patients with MDD to accurately assess their emotions numerically gives us an opportunity to be persuaded through an emotion measurement software FaceReader to see if participants’ facial emotions could show better differences between healthy people and patients suffering from MDD. There are studies in which facially expressed emotional responses to basic food tastes are reported (Greimel et al., 2006; Zeinstra et al., 2009; Bredie et al., 2014). However, no work has been done to compare these responses to different imagined food tastes in patients suffering from MDD.

Some explanations in the literature for the different food tastes and possible induced emotions are given. According to Noel and Dando (2015), positive emotions correlated with enhanced “sweet” taste and negative emotions with decreased sweet taste. Emotional manipulation with conventional antidepressants that target the serotonergic and adrenergic systems impact the sweet, bitter and sour taste thresholds (Heath et al., 2006) because serotonergic receptors are involved in taste transduction (Huang et al., 2009). Greimel et al. (2006) reported that ratings of the sweet stimulant in particular were regulated in accordance with the quality of the emotion, with “joy” increasing and “sad” decreasing the pleasantness and sweetness of the sweet stimulus. It was reported that a bitter taste or an extremely salty or sour taste could induce stronger emotion in comparison with pleasant tastes (Steiner, 1979; Hu et al., 1999; Rozin, 2006; Zeinstra et al., 2009). According to Bredie et al. (2014) on comparing different food tastes, among the different expressed emotions “disgust” was the most intense because it has a strong and specific facial expression (Rozin and Fallon, 1987; Greimel et al., 2006). According to Horio (2003), negative responses to unpleasant food tastes are induced and expressed more strongly in comparison with positive responses and, according to Greimel et al. (2006), are less related to the emotional state of the person. However, our study showed that the emotional state of patients suffering from MDD leads to a lower intensity for all the expressed emotions in comparison with the control group, and that this is the main difference between depressed and non-depressed persons. It was reported that verbal expressions or other kinds of gestures may be more important in conveying information about distaste (Bredie et al., 2014). Less expressed emotion is not necessarily a predictor for the absence of emotion, it could be due to lack of reason to express, because of masking and control (Zeinstra et al., 2009), or due to the emotional process itself not eliciting sufficient facial activity to be interpretable by the observer (Tassinary and Cacioppo, 1992). However, in our study, the lower expression intensity for all the fixed emotions could be explained by the specific disease, MDD, which is characterized by a lower feeling of joy during food consumption.

## Conclusion

The results obtained by using the FaceReader technique showed typical characteristics of the emotional responses of patients suffering from MDD to different imagined food tastes (“sweet,” “salty,” “bitter,” “sour,” and “neutral”): patients with MDD expressed lower “happy” and “contempt” and higher “surprised” emotions, along with a higher negative valence mean, in comparison with the control group for all the tested basic taste of food (*p* ≤ 0.05). Evaluation of the emotional response to imagined “sour” and “salty” tastes using a Likert scale showed, on average, 22.1 and 23.2% lower acceptability (*p* ≤ 0.001), respectively, for patients with MDD in comparison with the control group. The findings of this study provide the additional data on food–associated emotion analysis of MDD patients and could be useful for the further development of the contactless method for early diagnosis of MDD.

## Author’s Note

The patent registered in the State Patent Bureau of the Republic of Lithuania entitled “System for the people’s early stage depressive disorder detection” (Document no. 6735; Type B; Application no. 2018 030; date of application, 11-10-2018; date of publication of application, 27-04-2020; date of publication of patent, 25-05-2020) results from the work reported in this manuscript.

## Data Availability Statement

The datasets presented in this article are not readily available because the data of patients information used to support the findings of this study are restricted by the Bioethics Committee (No. 04/2017) in order to protect patient’s privacy. Questions regarding the datasets should be directed to VA (virginija.adomaitiene@lsmuni.lt) or GJ (grazina.juodeikiene@ktu.lt).

## Ethics Statement

The studies involving human participants were reviewed and approved by the Regional Bioethics Committee (No. 04/2017). The patients/participants provided their written informed consent to participate in this study.

## Author Contributions

VA, GJ, EB, and VS: conceptualization, methodology, and resources. DC and DK: software and validation. VL, EM, and LJ: formal analysis. VL, LJ, DC, EM, and DK: investigation. DC: data curation and visualization. DC, LJ, EM, and EB: writing – original draft preparation. EB, VA, GJ, and VS: writing – review and editing. VA and GJ: supervision, project administration, and funding acquisition. All authors have read and agreed to the published version of the manuscript.

## Conflict of Interest

The authors declare that the research was conducted in the absence of any commercial or financial relationships that could be construed as a potential conflict of interest.

## Publisher’s Note

All claims expressed in this article are solely those of the authors and do not necessarily represent those of their affiliated organizations, or those of the publisher, the editors and the reviewers. Any product that may be evaluated in this article, or claim that may be made by its manufacturer, is not guaranteed or endorsed by the publisher.
